# A climate region classification for California’s warm season: apparent temperature clustering to support heat-health epidemiology

**DOI:** 10.1007/s00484-026-03200-w

**Published:** 2026-04-20

**Authors:** Marinelle Villanueva, Sydney Monte-Sano, Scott Sheridan, Michael Allen, Laurence Kalkstein, Michael Jerrett, David P. Eisenman

**Affiliations:** 1 Department of Environmental Health Sciences, Fielding School of Public Health, University of California, Los Angeles, Los Angeles, CA USA; 2 Center for Public Health and Disasters and Center for Healthy Climate Solutions, University of California, Los Angeles, Los Angeles, CA USA; 3 Department of Geography, Kent State University, Kent, OH USA; 4 Department of Geography and Environmental Planning, Towson University, Towson, MD USA; 5 Applied Climatologists, Inc., Marco Island, FL USA; 6 Division of General Internal Medicine and Health Services Research, David Geffen School of Medicine, Department of Community Health Sciences, Fielding School of Public Health, University of California, Los Angeles, Los Angeles, CA USA

**Keywords:** Climate, GIS, Meteorology, Spatial analysis

## Abstract

Climate classification enhances our understanding of regional climate patterns and enables a science-based framework for assessing environmental and public health relationships. Prior climate classification systems are limited in their ability to capture variation across dynamic subclimates, particularly in the context of complex topographical, elevation, and meteorological characteristics such as California, United States. In this study, we spatially classified climate regions in California during the warm season (May through September) in 2021 and 2022. We applied principal component analysis with k-means clustering algorithms to gridded data consisting of apparent temperature during the study period as a monthly time series. We then performed statistical and spatial analysis to delineate the geographical extent of climate regions with shared apparent temperature distributions. The results of this study include a statewide map of 30 warm season climate regions based on meteorological data characterized by homogenous temperature patterns that are distinct from one another. The climate regions demonstrate highly variable spatial patterns of heat exposures and often span across and within multiple county boundaries. The methods we present within a complex geography can be readily adapted to other regional settings and updated to other temporal periods. This study informs our understanding of regional climate patterns during the warm season and is applicable to the development of early warning systems and quantifying extreme heat impacts across statewide populations.

**Supplementary Information:**

The online version contains supplementary material available at 10.1007/s00484-026-03200-w.

## Introduction

Climate classification systems are crucial for assessing regional climate patterns and the effects on meteorology, environment, and health. Understanding the relationships between climate and human health outcomes can be greatly enhanced by examining them through the lens of homogenous climate regions. Our understanding of climate impacts remains limited without accounting for underlying regional climate conditions that shape the human body’s ability to adapt to regular heat exposure, known as heat acclimatization (Brown et al. 2022; Liu et al. 2022). The health effects of a given temperature can vary substantially between regions, leading to heterogeneity in temperature-related health risks across geographic and population groups (Anderson and Bell 2009; Vaidyanathan et al. 2019; Bell et al. 2024). Climate region classifications are thus necessary for analyzing climate implications on environmental conditions, the impacts on human health, and planning for climate change adaptation.

Climate patterns can be delineated into regional classifications to capture the spatial extent of subclimates that are meaningful and interpretable for various applications. Climate classifications at the global (Köppen 1936; Thornthwaite 1948; Trewartha and Horn 1980) and regional scales (Jacob et al. 2012; Feng et al. 2024) have been extensively implemented for applications in various fields, including climate change modeling (Jylhä et al. 2010; Li et al. 2023), epidemiology (Anderson et al. 2019; Singh et al. 2024), urban planning (Coutts et al. 2008; Aslam and Rana 2022), and agriculture (Tao et al. 2014; King et al. 2018). Although these classification systems are well-established and widely adopted, they are limited in their ability to accurately reflect spatial and seasonal variability across subclimates.

California, United States (US) encompasses a wide range of subclimates – from highland mountains to arid deserts, mediterranean coastal to inland, urban to rural regions – and is a particular hotspot for climate change impacts such as extreme heat (Lenihan et al. 2003). Broad-scale climate change, regional climate dynamics, and population growth can lead to considerable variation in the distribution of heat exposure and related health risks (Sheridan et al. 2012; Vahmani et al. 2019). Temperature and humidity shapes heat vulnerability (Willett and Sherwood 2010). Maximum temperature, in particular, tends to vary on finer spatial and temporal scales (Cordero et al. 2011). Apparent temperature, also known as the heat index or “feels like” temperature, is a common exposure metric used in epidemiological studies of heat and health associations in the US and California because it reflects physiological heat stress by combining air temperature and relative humidity (Basu et al. 2012; Anderson et al. 2013; Khatana et al. 2022).

Various rule-based and clustering approaches have paved the way for identifying climate regions. Among the most widely used global schema, the Koppen climate classification, is a top-down approach based on historical patterns of precipitation, temperature, and vegetation (Köppen 1936). The Koppen classification system is rule based in which subjectively defined thresholds of climate characteristics are used to classify areas into predetermined climate types. While the Koppen system is widely adopted for its interpretability, this classification system is static and unable to account for emerging or shifting climates in an era of change (Lasantha et al. 2022). Cluster-based and data-driven classification systems based on temperature, precipitation, and temperature distinguish climates that are internally more homogenous and externally more distinct than subjectively defined climate types (Netzel and Stepinski 2016). These approaches can complement one another to produce an intuitive and objective climate classification system that is more adaptable to shifting climates while capturing fine-scale patterns in meteorology (Lasantha et al. 2022).

Only one climate classification specific to the state exists: the California Energy Code (CEC). It is based on factors including energy use, temperature, and weather, though is primarily used to determine building energy efficiency standards (CEC 2024). Climate classification tailored specifically for heat-health analyses is lacking. Previous studies have found that associations of extreme heat and risk of hospitalization vary across CEC regions, suggesting differences in heat acclimatization (Joe et al. 2016; Guirguis et al. 2018; Schwarz et al. 2020). The CEC classification does not represent patterns of heat exposure and may not accurately capture their spatial distribution. These patterns change across seasons due to shifts in physiological regulation and adaptive behaviors such as use of air conditioning or cooling centers (Schwarz et al. 2020), highlighting the need for a more dynamic and health-relevant framework. Our study addresses this gap by developing a mapping product designed to improve climate region classifications for heat-health analyses during the warm season.

The objective of this study was to delineate climate regions in California during the warm season. We present an integrated approach to classify climate regions based on cluster, spatial, and statistical analysis of apparent temperature as a monthly time series. We use apparent temperature, an index combining maximum temperature and relative humidity, as our primary heat exposure metric. Given that climate patterns can vary between seasons, we specifically focus on the warm season (May through September) to enhance our understanding how heat exposures are spatially distributed. This study was conducted to inform the development of public health tools for community-level heat warning systems and assessments of historical heat-related health impacts in California, which are currently implemented to identify at-risk communities and support local heat preparedness policies and plans.

## Materials and methods

### Study area

This study was conducted in California, US during the warm season (May through September) in 2021 and 2022. The geographical coordinates are 32.53º–43.01º north latitude (°N) and 114.13º–124.48º west longitude (°W) with elevations ranging from approximately  -80 m (-200 ft) to 4,400 m (14,500 ft). In total, the study area covers approximately 424,000 km^2^ (164,000 mi^2^). California borders the Pacific Ocean and features myriad geographical landscapes, including coastlines, valleys, mountains, and deserts which shape diverse meteorological conditions across the state.

### Data

#### Gridded meteorological data

We obtained daily average apparent temperature across California from gridded meteorological data during the warm season in 2021 and 2022. The warm season was defined as May through September based on prior literature of heat-health associations in California (Basu et al. 2012). Monthly average maximum temperature and humidity were derived from a gridded reanalysis (31 km x 31 km) of atmospheric, land, and oceanic climate variables from the European Centre for Medium-Range Weather Forecasts ERA5 Climate Reanalysis (ECMWF 2022). We included all land-based grid cells in California and surrounding states to ensure comprehensive coverage of the state’s geographical extent. A total of 1252 grid cells were obtained which included 900 grid cells completely within California’s state boundary. For each grid cell, we derived apparent temperature as a composite index of maximum temperature and humidity to reflect how temperature feels to the human body more accurately than temperature alone (Steadman 1984).

#### Population count data

We derived population count data and generated zip code population-weighted centroids using a fine-resolution population dataset. Given that populations are unevenly distributed and zip codes often extend into uninhabited areas, population-weighted centroids better represent where most people live. While zip code boundaries comprehensively cover the geographic extent of the state, they lack accurate population count data. We obtained a zip code shapefile from the California State Geoportal in 2020 (California Department of Technology 2020). We calculated zip code total population counts and population-weighted centroids using 2020 EnviroAtlas dasymetric population data from the Environmental Protection Agency, a high-resolution (30 m x 30 m) grid of reallocated population data from 2020 US Census blocks based on land cover classifications (US Environmental Protection Agency 2022; Baynes et al. 2022; Cartagena-Colón et al. 2022). A detailed explanation of these methods for the calculation of the zip code population-weighted centroid and total population using dasymetric mapping is provided in *Supplementary Information 1*.

## Methods

### Principal component analysis

To identify climate divisions, we performed cluster analysis using daily mean apparent temperature values from the warm seasons in 2021 and 2022. Each grid cell was converted to a point with latitude and longitude information based on the geometric centroid. Grid values across the state were characterized as a time series of 364 daily apparent temperature values (2 years x 182 days per warm season). We applied principal component analysis (PCA) to reduce the dimensionality of the data set to 21 principal components, retaining all components that had an eigenvector of > 1.0. These 21 components were used as the input for k-means clustering analysis with randomized starting points, which grouped the grid points into climate regions based on similar temporal patterns. We applied the NbClust function in R to determine the optimal number of clusters which uses 30 different validity indices, offering a robust assessment driven by the data (Charrad et al. 2014). This analysis identified 14 clusters as the optimal number, among options ranging from 10 to 20 clusters, forming the initial set of climate divisions. This set of 14 clusters also was broadly spatially contiguous.

**Fig. 1:**
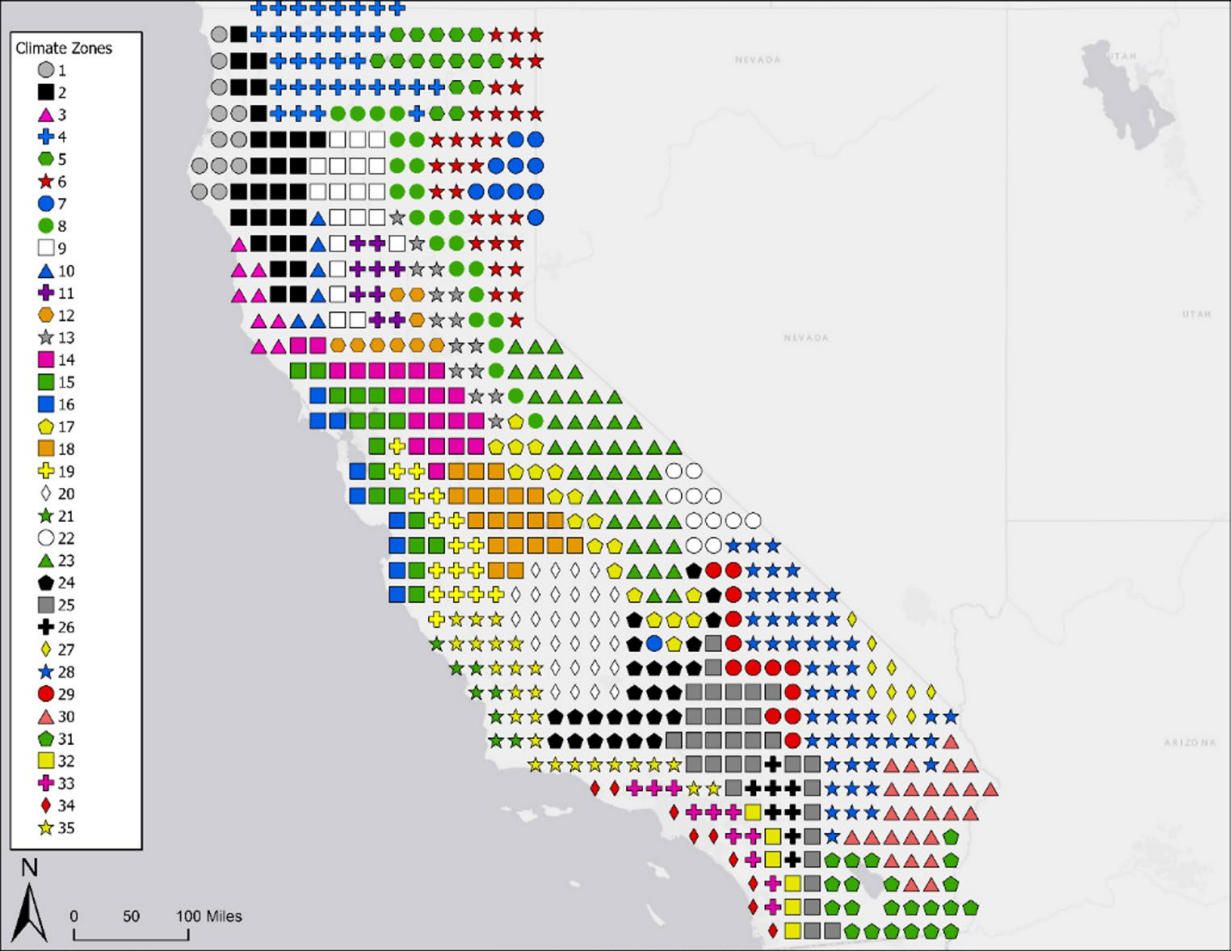
Map of the 35 climate zones identified via cluster analysis. Each symbol represents zones with similar patterns of mean monthly apparent temperature. The location of each point represents the centroid of a meteorological grid cell

After the initial 14 climate clusters were established, we conducted a second stage of clustering within each of those original clusters to identify subclusters of finer-scale climate patterns to help divide up the broader climate clusters. Using only the data that was categorized into each cluster in the first step, we applied the same approach using k-means clustering on the principal components to identify sub-clusters in each of the 14 initial clusters independently. In this second round, NbClust was used to determine the optimal number of subclusters with options ranging from 1 to 10 (whereby 1 signifies subclusters were not optimal). The results of Nbclust yielded one cluster for which no subclusters were identified, while for the other clusters, between 2 and 7 subclusters were identified. Collectively, this process identified a total of 35 distinct cluster points (Fig. 1).

### Spatial, demographic, and statistical analysis

**Fig. 2:**
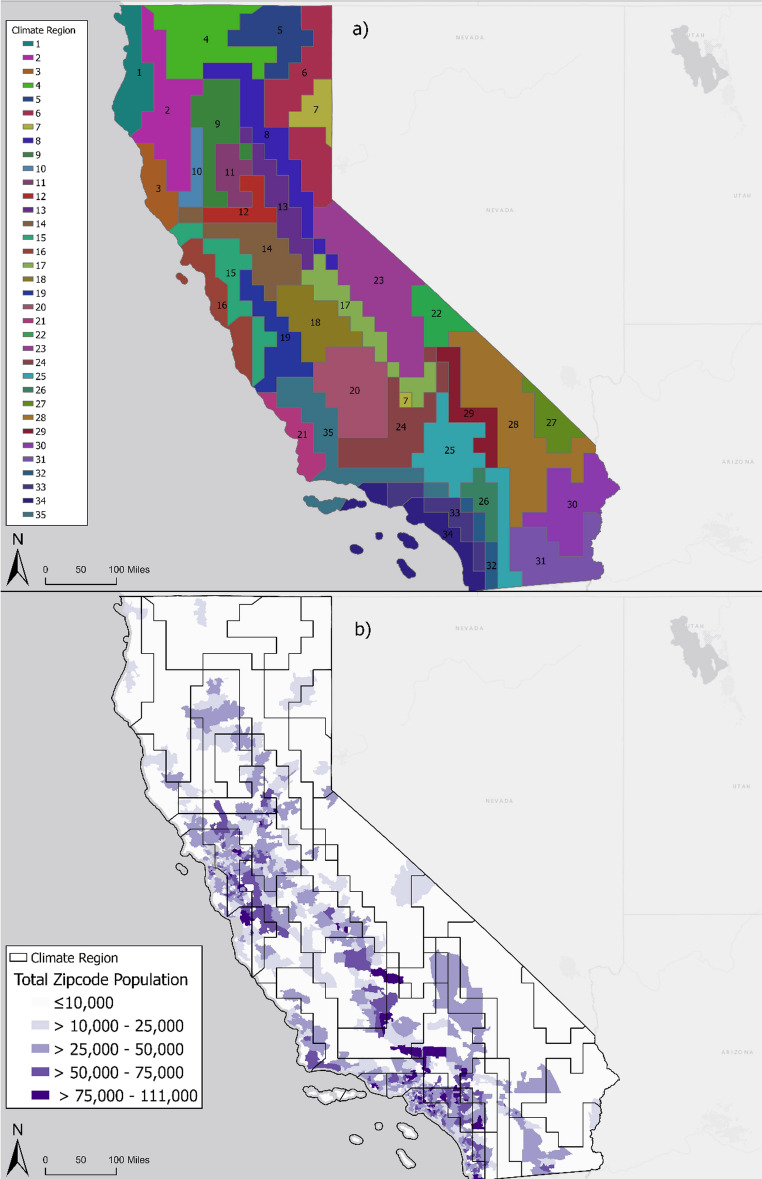
Map of the (**a**) 35 climate region classifications during the warm season (May through September) from 2021 and 2022 in California and (**b**) estimated total population in zip codes

We converted the cluster points to spatial polygons to delineate the area for each of the 35 climate regions. Using Thiessen (Voronoi) polygons, we developed geometric polygons to partition the space around the points. Thiessen polygons are a spatial technique used to divide space around points, such that any location inside the polygon is closer to the point with the same classification than other points (Mayya and Rajan 1996). This analysis created a map across California outlining 35 polygons (Fig. 2a), delineating climate regions with similar climate patterns.

Five of the 35 climate regions appeared to be isolated clusters which warranted assessment of the total population in each climate region (Fig. 2b). Although the cluster analysis guided our delineations, we consolidated highly fragmented or sparsely populated regions to improve interpretability and maintain adequate sample size and statistical power for future epidemiology analyses. We assigned zip codes to climate regions using the population-weighted centroid and summed the total population of zip codes in each climate region. We identified fragmented regions with relatively small populations sizes, defined as regions with fewer than 11,000 people (bottom 10th percentile), as eligible to be combined with a nearby climate region with similar meteorological and geographical characteristics (Fig. S1 in Supplementary Information).

Finally, we performed further spatial and statistical analysis to consolidate the initial 35 regions to 30 distinct regions. For each given climate region, we computed the difference in the mean apparent temperatures in each month relative to all other regions. We performed Wilcoxon Signed-Rank tests between pairs of regions to assess whether the mean monthly temperatures were significantly different using an α = 0.1. Wilcoxon Signed-Rank Tests indicated that the differences in the mean monthly temperatures between selected pairs of regions were not statistically significant (Table 1). Neighboring regions show statistically significant differences in the mean monthly temperatures, for instance regions 30 and 28 and regions 30 and 27 (Table 1).

For the fragmented regions with small population sizes, we identified *selected pairs* of nearby climate regions in which the (1) mean monthly apparent temperatures did not significantly differ, and (2) topographical and elevation characteristics were similar. Selected pairs of regions, which included a fragmented region with a small population size, were combined as follows: regions 5 and 4, 7 and 8, 7 and 17, 22 and 24, 27 and 29, and 30 and 31. Spatial assessments confirmed the selected pairs shared similar elevation and topographic characteristics. The regions for these pairs were then combined. The majority of region 7 was combined with Region 8 since these regions are adjacent and had similar temperature patterns. We combined only the isolated cluster for region 7 with region 17 because they are contiguous and have smaller differences in monthly temperature than regions 7 and 24.

We combined the boundaries for five of the selected pairs of climate regions meeting the eligibility requirements, typically corresponding to nearby or contiguous pairs of regions with the smallest relative difference in the mean monthly apparent temperature. The final classification of 30 warm season climate regions reflected a combination of data-driven methods and spatial assessments.

We utilized R programming v4.3.2 and ArcGIS Pro v3.2.0 for cluster, statistical, and spatial analysis.

**Table 1:** Absolute differences of mean monthly temperatures between two climate regions and results from Wilcoxon Signed-Rank tests

Region Pairing	May	June	July	August	September	*p*-value ^A^
	(Δ°C)	(Δ°C)	(Δ°C)	(Δ°C)	(Δ°C)	
4 and 5	0.5	0.2	0.4	0.0	0.5	0.62
7 and 8	0.2	0.2	0.8	0.7	0.0	0.31
7 and 17 ^B^	3.1	2.6	1.5	1.8	3.0	0.06
22 and 24	0.3	1.4	1.3	0.8	0.3	0.19
27 and 29	0.1	0.2	0.1	0.2	0.1	0.19
30 and 31	0.0	0.1	0.1	0.5	0.8	0.31
30 and 28 ^C^	2.6	2.4	2.2	2.8	3.1	0.06
30 and 27 ^C^	5.7	5.3	5.2	5.7	5.7	0.06

^A^ Results from Wilcoxon Signed-Rank tests, assessed at an α = 0.1

^B^ Only the isolated cluster for region 7 was combined with region 17, and the rest of region 7 was combined with Region 8

^C^ Comparison of monthly apparent temperature for regions that were not combined (30 and 28, 30 and 28)

### Climate region descriptions

We described each of the 30 climate regions to represent the location and temperature climate. We use the California Fourth Climate Change Assessment Regions as the locational descriptor given their relevance to regional climate action planning. There are nine climate change assessment regions that divide the state to analyze localized impacts, given that climate adaptation planning and program implementation often occur at local and regional scales (CA Natural Resources Agency 2022). We indicate climate change assessment region(s) that a climate region overlaps with at least 25% of the spatial area. Temperature climates are categorized by mean monthly apparent temperature as follows: coolest (50–60 °F), cool (60–70 °F), moderate (70–75 °F), warm (75–80 °F), hot (80–85 °F), and hottest (≥ 85 °F). For each region, we identify the modal temperature category across the study period; if a region’s temperature distribution spanned two categories, then both are reported.

## Results

### Climate region classifications

**Fig. 3:**
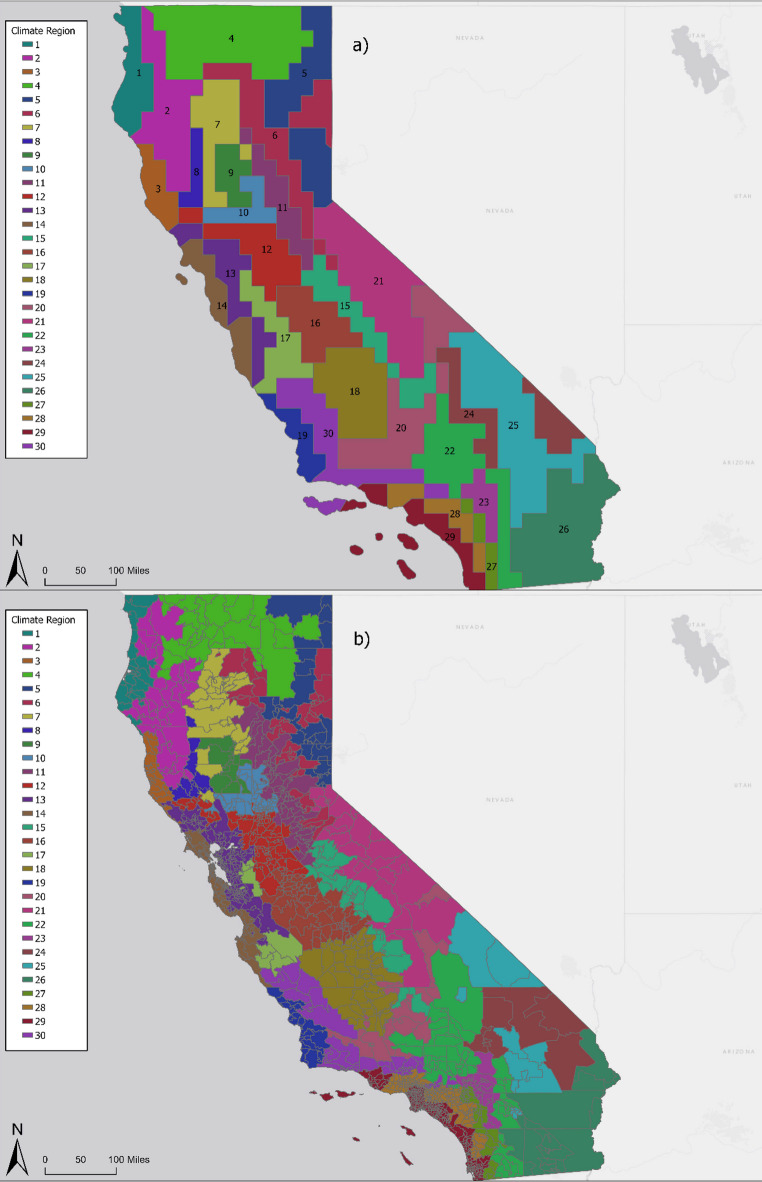
Map of the (**a**) 30 climate region classifications during the warm season (May through September) from 2021 and 2022 in California and (**b**) climate regions assigned to zip codes

We present a map of the warm season climate regions in California with a total of 30 regions (Fig. 3a) and are additionally presented as zip codes assigned to a climate region via the population-weighted centroid (Fig. 3b). The figure illustrates the highly variable spatial distribution of heat exposures. Summed across the zip code populations within climate regions, regional population counts range from 29,609 in region 3 to 11,378,685 in region 29 with an average population of approximately 1.3 million (Table 2). Across the regions we identified, mean monthly apparent temperatures during the study period ranged from 9.4 °C (49.5ºF) in region 5 to 35.5 °C (93.9ºF) in region 31.

**Fig. 4:**
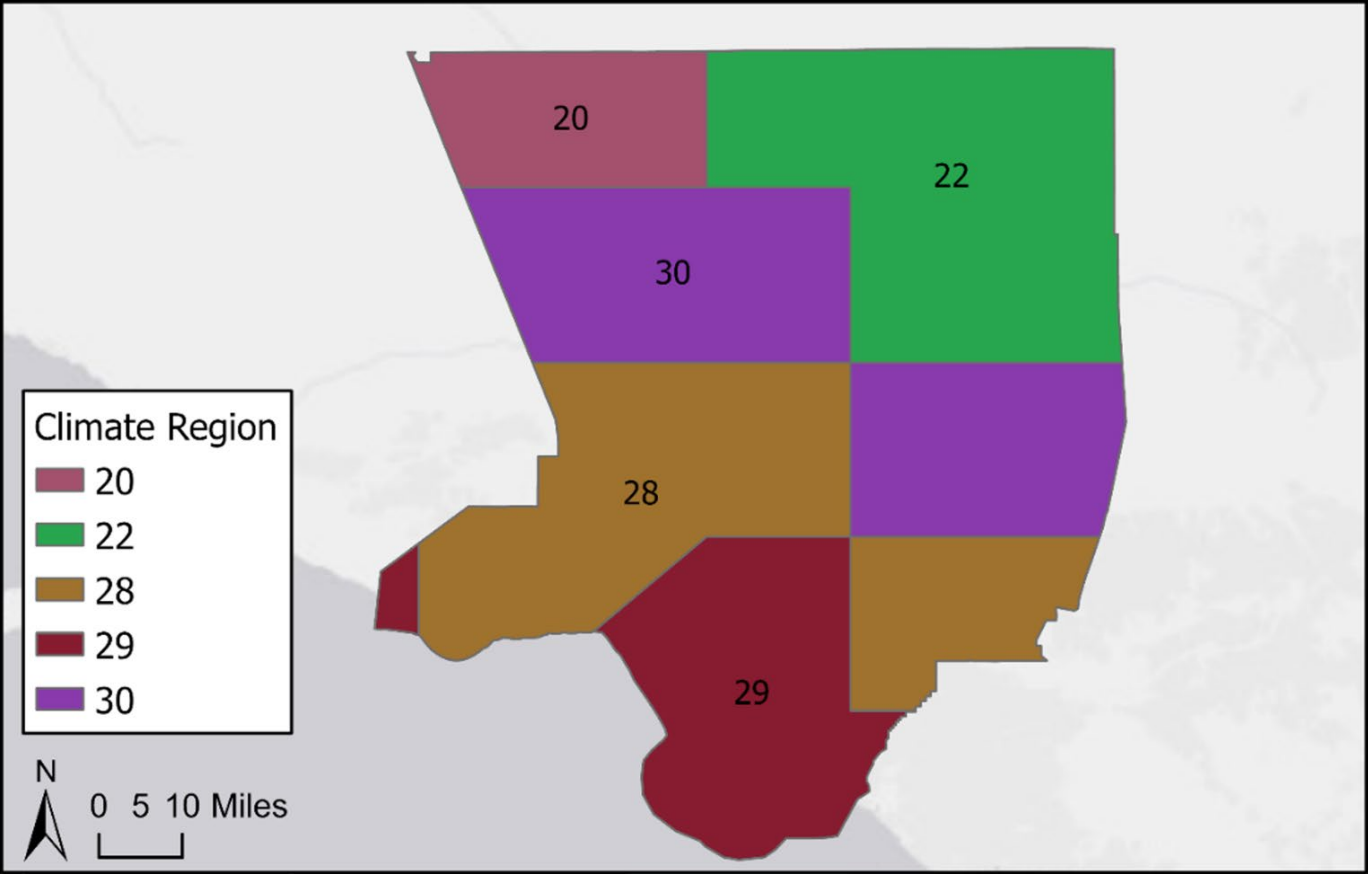
Climate regions in Los Angeles County, California based on 30 warm season climate region classifications

Spatial assessments demonstrated that regions generally followed patterns in topography and elevation. For instance, in regions 28 and 29, coastal areas cooled by ocean breezes are partitioned from inland areas with warmer temperatures. Mountainous regions in the northernmost parts of California with high elevations and forested terrains are distinguished between lower elevation valleys. Fine-scale heterogeneity exists within shared geographies, for example, where deserts in the southeastern parts of the state are distinguished as separate climate regions from one another. This variation is apparent at the county level, such as in Los Angeles County, where five climate regions are identified within a single county (Fig. 4). These climate regions represent the distinct and fine-scale variation of sub-climate partterns during the warm season across the state.

Each region is described in Table 2 to represent the climate change assessment region and apparent temperature pattern. Climate change assessment regions intersect several temperature patterns within our climate regions, such as in the North Coast. Similarly, individual climate regions frequently span more than one climate change assessment region, particularly regions 7–10 and 23–27.

**Table 2:** Descriptions and estimated total population in the 30 warm season climate regions in California

Region	Description	Total population
1	North Coast Coolest	156,557
2	North Coast Mountain Cool	89,006
3	North Coast Cool	29,609
4	North East Plateau Coolest	55,841
5	Sierra Nevada Mountains Coolest	50,705
6	Sierra Nevada Mountains-Sacramento Valley Cool	81,623
7	Sacramento Valley Moderate	362,991
8	North Coast-Sacramento Valley Moderate	53,073
9	Sacramento Valley Moderate-Very Warm	55,991
10	Sacramento Valley Moderate-Warm	1,123,510
11	Sierra Nevada Mountains-Sacramento Valley Moderate	504,172
12	San Joaquin Valley-Sacramento Valley Moderate	2,357,678
13	San Francisco Bay Area Cool	5,554,712
14	San Francisco Bay Area-Central Coast Coolest	2,613,283
15	Sierra Nevada Mountains Moderate	122,437
16	San Joaquin Valley Warm-Hot	1,511,043
17	North Central Coast-San Joaquin Valley Moderate	241,159
18	San Joaquin Valley Hot	1,495,011
19	Central Coast Cool	451,443
20	San Joaquin Valley-Sierra Nevada Mountains Moderate	88,862
21	Sierra Nevada Mountains Coolest	59,548
22	Inland Desert Hot	1,323,832
23	Los Angeles-Inland Desert Warm	413,345
24	Inland Desert-Sierra Nevada Mountains Hot	46,364
25	Inland Desert-Sierra Nevada Mountains Hottest	251,095
26	Inland Desert Hottest	237,606
27	San Diego-Los Angeles Warm	1,079,402
28	Los Angeles-San Diego Warm	6,766,869
29	Los Angeles-San Diego Moderate	11,378,685
30	Central Coast-Los Angeles Moderate	98,4304

### Regional variation in heat-health risk

The climate regions described in this study were incorporated into our analysis to develop a statewide, community-level early warning system and maps of historical heat-health impacts in California. We use the climate regions to estimate the average maximum apparent temperature as baseline (counterfactual) heat for each day in respective zip codes to identify heat days in which elevated health impacts are observed. Using emergency department visits in California (2008–2018), we rank heat days according to excess heat-related emergency department visits at the neighborhood level and estimate the temperature associated with elevated health impacts. Impact levels in heat days are ranked as mild, moderate, high, or severe. This approach allows us to account for spatial and temporal differences in temperature patterns and accompanying heat acclimatization between populations. More information regarding the early warning system is accessible at the following link: https://calheatscore.calepa.ca.gov. Our results for historical heat-health impacts can be accessed for each zip code and county in California via our interactive website: https://experience.arcgis.com/experience/aa2b2ec9df1d494f9348c5c1941aa497/page/Map.

**Fig. 5:**
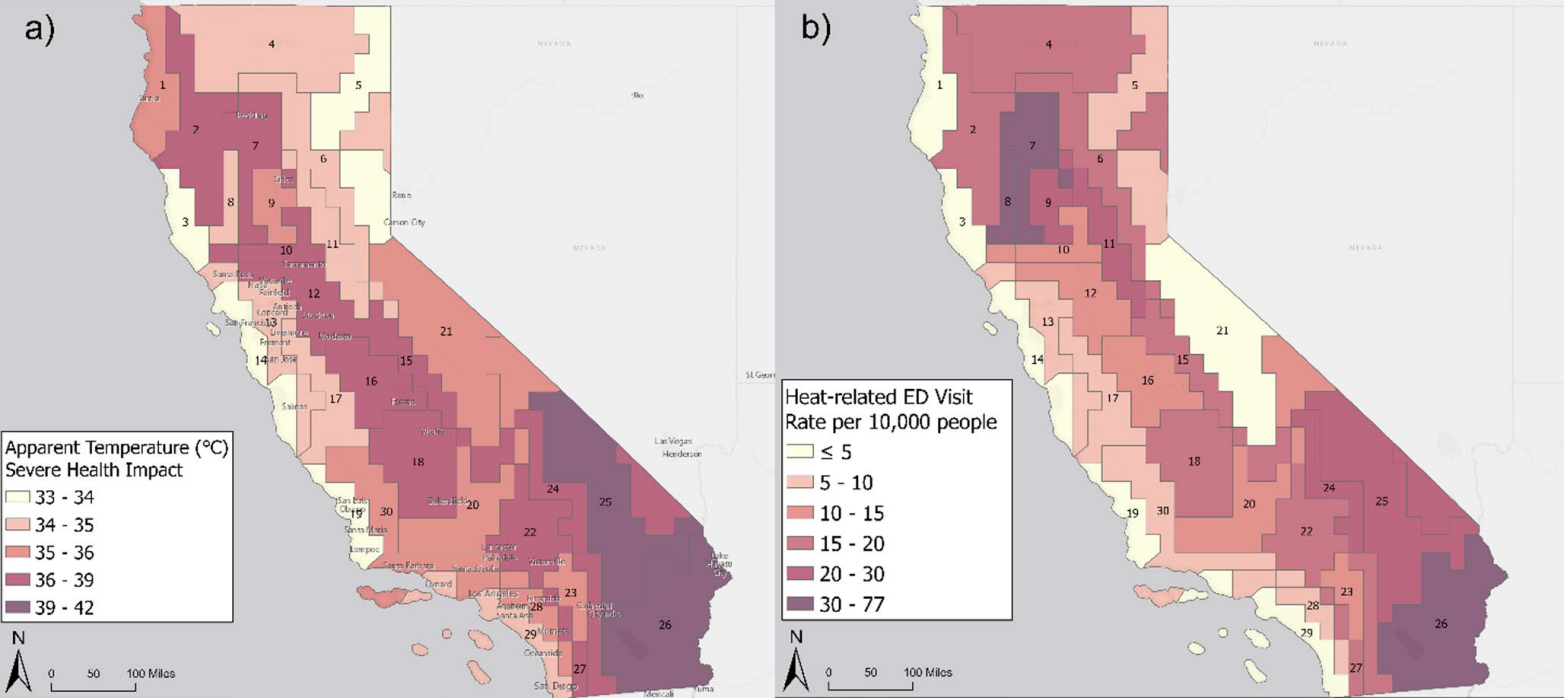
Climate region distribution of (**a**) maximum apparent temperature and (**b**) annual rate of excess heat-related emergency department visits per 10,000 people associated with severe health impacts (2008–2018)

To illustrate how heat-health impacts vary within and across regions, we describe the climate region distribution of maximum apparent temperature (Fig. 5a) and the annual rate of excess heat-related emergency department visits per 10,000 people (Fig. 5b) associated with severe impacts between 2008 and 2018. Figure 5a shows significant heterogeneity of health impacts associated with heat exposure, with severe impacts occurring at lower temperatures in coastal and mountain regions and higher temperatures in inland desert regions. In contrast, heat-health relationships are more similar among zip codes within the same region than in neighboring regions. Regions 30 and 20, for example, are adjacent in Los Angeles county (Fig. 4). We observed severe impacts in region 30 zip codes (91351 and 91350) at 36.5 °C and 35.3 °C, compared with 38.3 °C and 38.2 °C in region 22 zip codes (93550 and 93552). Coastal populations (region 30) show vulnerability at lower temperatures, while inland populations (region 22) appear to be more acclimatized to higher heat exposure. Within-region heterogeneity is observed in region 30 where we estimated an annual rate of 7.3 and 9.4 in zip codes 91350 and 91351 (region 30), which potentially reflects zip code-level demographic differences. Rates were similar in zip codes 93550 and 93552 (region 22) zip codes, suggesting similar within-region heat acclimatization.

## Discussion

In this study, we utilize maximum temperature and relative humidity data in California to spatially partition warm season climate regions statewide. California’s highly variable geographic, elevation, coastal, and topographic features delineate subclimate areas. By applying this analysis within a climatologically complex state, we present an integrated approach that is adaptable to changes in climate and diverse geographical characteristics. We map and classify 30 climate regions which capture homogenous climate patterns with distinct apparent temperature distributions. Heterogeneity within counties indicates fine-scale variability that is typically obscured when using county-level units of analyses. Individual climate regions often intersect multiple climate change assessment regions, further underscoring the need for a science-based framework that accounts for varying climate patterns beyond existing boundaries. We additionally show how the relationship between heat exposure and health impacts substantially varies across climate regions.

Our study demonstrates that incorporating climate regions to account for the distribution of temperature patterns can enhance heat-health analyses that enable community-level early warning systems and estimates of health impacts related to heat exposure. Prior research in California has identified heat exposures as a risk to human health, which is often assessed at the county level (Ebi et al. 2004; Basu et al. 2008; Baker and Sturm 2024). County-level estimates, however, are unable to capture spatial heterogeneity at finer scales and are not generalizable across populations that experience different degrees of temperature distributions. Heat-health associations at the local scale, such as zip codes, may be biased without adjusting or stratifying by climate region. Our findings align with a study in San Diego County which observed that hospitalization rates among coastal populations increase at lower temperatures compared to inland populations, suggesting that heat vulnerabilities spatially vary between climate regions (Guirguis et al. 2018). Community-level assessments of how temperature thresholds correspond to elevated heat risk or what characteristics may modify susceptibility to heat exposure are critical for developing effective heat warning systems and identifying priority areas for allocating health-protective resources (Gronlund 2014; Schwarz et al. 2021). Climate classifications therefore enable smaller-scale assessments that can help inform local and regional adaptation planning.

### Comparison with prior climate classifications

Existing climate maps lack detailed and comprehensive partitions of climate. For instance, the National Weather Service’s climate map of the temperature outlook during the warm season (July through August) uses only temperature data to determine the likelihood of abnormal temperatures and classifies only two levels in California, yet this does not necessarily depict the geographic extent of climates (NWS 2025). The Koppen Climate Classifications are defined by subjectively determined meteorology thresholds for climate types that are unable to adapt to changes in climate; therefore, this classification is not suitable for future analyses requiring climate data that capture meteorological variability of more recent conditions (Lasantha et al. 2022). The California Energy Code (CEC) climate classifications were designed to capture energy consumption patterns based on annual meteorological data from stationary weather stations in 16 pre-selected cities (CEC 2024). The CEC climate regions do not represent warm season climates, and the methods used to delineate their boundaries are insufficiently documented and lack a robust meteorological basis. Therefore, our study fills this data gap by comprehensively assessing the spatial and temporal meteorological patterns and mapping warm season climate regions that support future climate, environmental, and health analyses.

Our approach objectively defines climate regions using clustering, spatial, and statistical techniques to identify meteorologically homogenous climates. Meteorological data includes apparent temperature, which reflects how heat is experienced by the human body, unlike other classification systems typically based on one meteorological variable. We analyze meteorological data in 2021 and 2022 to ensure relevance to recent climate conditions and to support future epidemiological analyses of heat and health. Our analysis specifically identifies warm season climate regions with the aim to better disaggregate the variability in heat acclimatization across a statewide population, which is influenced by local patterns of temperature and humidity (Racinais et al. 2019). The techniques presented in this analysis are adaptable and allow for climate region classification analyses in other regions or temporal periods.

We acknowledge some limitations. First, the temporal unit of analysis is limited to mean monthly apparent temperature for the warm season over two years. Given the large number of grid points and the mass of data, our cluster analysis produced more stable results during a two-year span. We doubt there would be important changes if another year or two were included. Second, we consider only the warm season since our purpose was to delineate heat-related human health variability across the state. Future research is needed to classify climates during the cold season to assess whether these climate patterns differ spatially. This methodology is certainly suitable for cold-season classification. As temperature may vary within a month or at even smaller spatial resolutions, further studies may consider finer scale variability to enrich data-driven climate clustering outputs.

### Research and policy applications

This study proposes a data-driven climate regionalization approach to develop maps of climate patterns during the warm season. Climate region data provided by this study can be assigned to spatial units of analysis of varying resolutions, such as zip codes as depicted in this manuscript, and applied to statistical associations between apparent temperature and administrative health data. This allows investigators to adjust for the extent to which susceptibility in exposure to heat varies in relation to heat acclimatization and permits the development of weather-health algorithms that are climate region-specific. It is reasonable to assume that, if socioeconomic characteristics are kept constant, then human health effects associated with heat will be similar within any single climate region. Without incorporating data for climate regions, research is limited by the assumption that the effect of heat exposure on health is uniform statewide and thus may result in biased estimations. Therefore, this product provides a science-based framework that enhances health assessments for the effects of heat on human health despite variability across populations.

Our climate region data has already been applied in analyses of health impacts related to exposure to extreme heat toward the development of a heat warning system and mapping historical heat-health impacts in California (OEHHA and UCLA 2025; UCLA and OEHHA 2025). These novel mapping products inform legislators, healthcare practitioners, community organizations, and individuals in planning for resource allocations and risk communication strategies to effectively protect populations from heat, where targeted interventions may vary from one climate region to another. Such analyses are strengthened through the adjustment for subclimates. Future research should apply these methods in other regional settings that are vulnerable to heat to enhance our understanding of climate-driven impacts.

## Conclusion

While prior climate regionalization systems have been well-established, season-specific and meteorology-based classification systems offer a robust framework for identifying and classifying climates for health and environmental analyses. Our approach integrates temperature and humidity data using clustering, spatial, and statistical techniques to delineate novel warm season climate region classifications that are crucial for interpreting, analyzing, and communicating climate-related impacts. According to the results obtained, California has 30 unique climate regions during the warm seasons of 2021 and 2022. These subclimates are evident across the state and county scales and are shaped by varying meteorological and geographical dynamics. We illustrate how the relationship between heat and human health impacts varies across climate regions. For climate adaptation plans based on environmental impacts on health, it is necessary for researchers to account for climate acclimatization using data-driven approaches. Analyses at larger geographic scales that do not account for climate regions can obscure this variability and may not estimate accurate health associations. The results of this study can be applied to support assessments of climate and environmental health impacts and to analyze the spatial climate dynamics in other regions of the world or temporal periods.

## Supplementary Information

Below is the link to the electronic supplementary material.

Supplementary Material 1

## Data Availability

The datasets generated during and/or analyzed during the current study are available in the Climate Regions repository, https://github.com/CalHeatScore-UCLA-modeling/ClimateRegions.
