# Supplementary material for: A climate region classification for California’s warm season: apparent temperature clustering to support heat-health epidemiology
Source: Int J Biometeorol. 2026 Apr 20;70(5):134. doi: 10.1007/s00484-026-03200-w (PMC13095919; doi:10.1007/s00484-026-03200-w)
Supplement: Supplementary file 1 — Supplementary Material 1 [file 484_2026_3200_MOESM1_ESM.docx]

**Title: A Climate Region Classification for California’s Warm Season: Apparent Temperature Clustering to Support Heat-Health Epidemiology**

**Authors:** Marinelle Villanueva ^A,F^, Sydney Monte-Sano ^B^, Scott Sheridan ^C^, Michael Allen ^D^, Laurence Kalkstein ^E^, Michael Jerrett ^A,F^, David P. Eisenman ^B,F^

1. Department of Environmental Health Sciences, Fielding School of Public Health, University of California, Los Angeles, CA 90095; email: msvillanueva@ucla.edu, mjerrett@ucla.edu
2. Center for Healthy Climate Solutions, University of California, Los Angeles, CA 90095; email: sydneymontesano@ucla.edu, msvillanueva@ucla.edu, mjerrett@ucla.edu, deisenman@mednet.ucla.edu
3. Department of Geography, Kent State University, Kent, Ohio 44242 email: ssherid1@kent.edu
4. Department of Geography and Environmental Planning at Towson University, Towson, Maryland 21252; email: mallen@towson.edu
5. Applied Climatologists, Inc., Marco Island, FL 34145; email: larryk@miami.edu
6. Division of General Internal Medicine and Health Services Research, David Geffen School of Medicine; Department of Community Health Sciences, Fielding School of Public Health, University of California, Los Angeles, CA 90095; email: deisenman@mednet.ucla.edu

**Correspondence:** Marinelle Villanueva, msvillanueva@ucla.edu

**Table of Contents**

1. **Supplementary Information 1.** Methods for using dasymetric population data to calculate total population and population-weighted centroids for zipcodes in California
2. **Supplementary Fig. S1** Total population in the initial 35 climate regions

**Supplementary Information 1.** Methods for using dasymetric population data to calculate total population and population-weighted centroids for zipcodes in California

The EnviroAtlas dasymetric population dataset, provided by the US Environmental Protection Agency, is a high-resolution (30m x 30m) grid derived from 2020 Census block data which reallocates population counts based on land cover classification (US Environmental Protection Agency 2022; Baynes et al. 2022). This approach uses supervised learning to exclude non-habitable areas such as open water, wetlands, and steep slopes (>25%), while classifying population estimates in developed and vegetated areas. Using this gridded dasymetric population data, we calculated zipcode population-weighted centroids based only on land areas where people live (Cartagena-Colón et al. 2022). We first performed a spatial join based on the largest overlap between the dasymetric population grid cells and 2020 California zipcode boundaries. Total population counts in each zipcode were derived as the sum of all dasymetric population grid cells joined to a zipcode. We then converted the population grid into points using the geometric center of each grid cell to obtain latitude and longitude information. Population-weighted centroids were computed as the weighted average of all point latitudes and longitudes within a zipcode, weighted by the grid cell population count.

**References**

Baynes J, Neale A, Hultgren T (2022) Improving intelligent dasymetric mapping population density estimates at 30&thinsp;m resolution for the conterminous United States by excluding uninhabited areas. Earth Syst Sci Data 14:2833–2849. https://doi.org/10.5194/essd-14-2833-2022

Cartagena-Colón M, Mattei H, Wang C (2022) Dasymetric Mapping of Population Using Land Cover Data in JBNERR, Puerto Rico during 1990–2010. Land 11:2301. https://doi.org/10.3390/land11122301

US Environmental Protection Agency (2022) EnviroAtlas - 2020 Dasymetric Population for the Conterminous United States v1.1 - Catalog


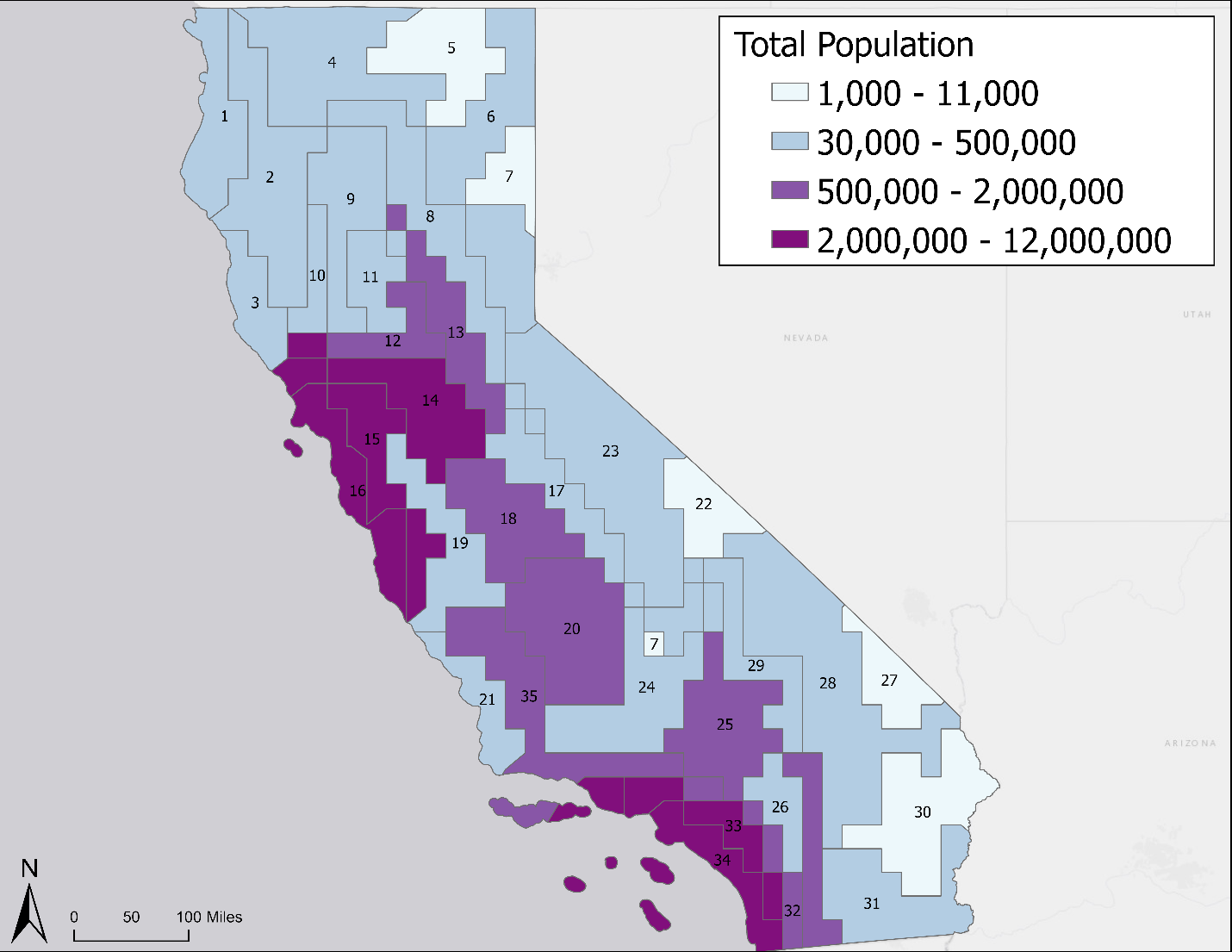


**Supplementary Fig. S1** Total population in the initial 35 climate regions
